# Health Concerns of Youths From Historically Marginalized Communities During the Postacute Phase of COVID-19

**DOI:** 10.1001/jamanetworkopen.2025.0837

**Published:** 2025-03-14

**Authors:** Melissa A. Jones, Margaux Jones, Kathleen M. Mazor, Benjamin P. Linas

**Affiliations:** 1 Harvard College, Cambridge, Massachusetts; 2 Tufts University School of Medicine, Boston, Massachusetts; 3 Division of Health Systems Science, Department of Medicine, UMass Chan Medical School, Worcester, Massachusetts; 4 Section of Infectious Diseases, Department of Medicine, Boston University School of Medicine and Boston University School of Public Health, Boston, Massachusetts

## Abstract

This survey study uses text message surveys to explore the behaviors, perspectives, and health concerns of youths from historically marginalized racial and ethnic groups in Boston, Massachusetts, during the postacute phase of the COVID-19 pandemic.

## Introduction

The COVID-19 pandemic highlighted substantial disparities in outcomes, mental health risks, and access to resources among youths from historically marginalized racial and ethnic groups.^1,2,3^ These disparities were compounded by limited research and outreach efforts to understand their needs. We engaged community members to help us explore the behaviors, perspectives, and health concerns of historically marginalized youths in Boston, Massachusetts, during the postacute phase of the COVID-19 pandemic using a text message–based survey.

## Methods

Data collection was conducted from April through July 2023. This survey study was approved by the Boston University Medical Center institutional review board. This report adheres to the AAPOR reporting guideline for survey studies.

Youths aged 14 to 24 years who self-identified as being from historically marginalized racial and ethnic groups and Boston-area residents were recruited through in-person events and social media. Participants self-reported their eligibility through a screening tool. After confirming eligibility on screening and providing assent for study enrollment, participants completed a questionnaire, providing their phone number and demographic information, including race and ethnicity.

Participants received 1 survey per week, over 5 weeks, via text message. We collaborated with local youths from historically marginalized communities to develop and refine survey content. Responses were analyzed using descriptive statistics and conventional qualitative content analysis by 2 coders (M.A.J. and M.J.). Responses were manually screened for quality assurance. Additional details are shown in the eMethods in Supplement 1.

## Results

In total, 49 eligible youths were enrolled and completed 153 surveys over the study period (Table 1). Most participants identified as female (71%), African American or Black (41%), or Asian (35%), with 27% identifying as Hispanic.

**Table 1.** Participant Demographics

Characteristic	Participants, No. (%) (N = 49)
Sex	
Female	35 (71)
Male	11 (22)
Other	2 (4)
No response or skip	1 (2)
Race	
African American or Black	20 (41)
American Indian or Alaska Native	2 (4)
Asian	17 (35)
White	4 (8)
Biracial or multiracial	3 (6)
Other^a^	2 (4)
No response or skip	1 (2)
Hispanic origin	
No	36 (74)
Yes	13 (27)
Health insurance or alternative coverage	
No	3 (6)
Yes	38 (78)
Unsure	7 (14)
No response or skip	1 (2)
Participant education level	
Less than a high school diploma	21 (43)
High school degree or equivalent (eg, General Educational Development)	10 (20)
Some college, no degree	12 (25)
Associate degree (eg, AA, AS)	0
Bachelor’s degree (eg, BA, BS)	4 (8)
Master’s degree (eg, MA, MS, Med)	2 (4)
Doctorate or professional degree (eg, MD, DDS, PhD)	0
Parent education level	
Less than a high school diploma	11 (22)
High school degree or equivalent (eg, General Educational Development)	11 (22)
Some college, no degree	4 (8)
Associate degree (eg, AA, AS)	6 (12)
Bachelor’s degree (eg, BA, BS)	7 (14)
Master’s degree (eg, MA, MS, Med)	5 (10)
Doctorate or professional degree (eg, MD, DDS, PhD)	5 (10)
School type	
Public	42 (86)
Private	1 (2)
Charter	2 (4)
Other	0
Not yet in high school	4 (8)
Concerned about finances in the last 12 mo	
Rarely	16 (33)
Sometimes	21 (43)
Most of the time	12 (24)
Household size	
≤2	10 (20)
3 or 4	16 (33)
≥5	10 (20)
No response or skip	13 (27)
Vaccinated against COVID-19?	
No	5 (10)
Yes	44 (90)
Prior COVID-19 infection?	
No	21 (43)
Yes	28 (57)

^a^ Options for race were defined by the investigator. The other category for race did not include specific races. It was not an aggregation of other responses, but rather a stand-alone option.

Forty-one of 49 participants (84%) completed the week 1 survey (Table 2). Respondents most commonly reported using the internet (49%) as their primary source of COVID-19 information, followed by news outlets, parents or guardians, and social media (16%-20%); 68% of respondents reported having a vaccinated parent or guardian. Parental influence on vaccination decisions, ranging from low to high, was evenly distributed across categories. Some respondents reported parental concern about boosters and general mistrust of vaccines, often associated with health and religious factors.

**Table 2.** Survey Responses

Questions and responses	Respondents, No. (%)^a^	
	2020 and 2021	2023
Week 1 survey questions: Influence on perception of COVID-19 vaccination (n = 41 respondents)		
Who was the first person or outlet you turned to for information about COVID-19?		
Internet (when in doubt, ask Google!)	20 (49)	NA
News outlets	8 (20)	NA
Parents or guardians	8 (20)	NA
TikTok, Instagram	8 (20)	NA
Friends	2 (5)	NA
Teachers	2 (5)	NA
Twitter	1 (2)	NA
Others	2 (5)	NA
Take a guess, how many of your friends have been vaccinated against COVID-19?		
All	11 (27)	NA
Majority	18 (44)	NA
About half	1 (2)	NA
Few	9 (22)	NA
None	1 (2)	NA
Prefer not to answer	1 (2)	NA
Do you have a parent or guardian who has NOT been vaccinated against COVID-19?		
Nope	28 (68)	NA
Yep	6 (15)	NA
Don’t know	5 (12)	NA
Prefer not to answer	1 (2)	NA
No response	1 (2)	NA
How much did your parent(s) or guardian(s) influence your decision to get or not to get vaccinated?		
Very	10 (24)	NA
Somewhat	9 (22)	NA
A little	10 (24)	NA
Not at all	10 (24)	NA
Prefer not to answer	2 (5)	NA
What concerns did they express?^b^		
Were against additional COVID-19 vaccinations or boosters	6 (15)	NA
Were concerned about long-term health effects or general mistrust of vaccines	7 (17)	NA
Week 2 survey questions: Influence on perception of COVID-19 vaccination (n = 27 respondents)		
If you can, define the top issue you’d most like to see addressed to improve the health, resilience, and well-being of your family and your community^b^		
Spaces to exercise	2 (7)	NA
Increased access to health care or health insurance	5 (19)	NA
Mental health resources	2 (7)	NA
Trans-affirming care	2 (7)	NA
Not informative or other	10 (37)	NA
No response or skip	7 (26)	
Are you part of any groups or organizations that you feel help you be healthy? What are these groups or organizations? (Please respond by listing them out.)^b^		
A part of no organization or no response	16 (59)	NA
A part of 1 organization	7 (26)	NA
A part of more than 1 organization	4 (15)	NA
We Got Us is a community organization that aims to support youth. What could we do (programs or activities) to help you lead a healthy life?^b^		
Community events or programming	10 (37)	NA
Educational sessions or workshops	9 (33)	NA
Free mental health resources	2 (7)	NA
Not informative or other	6 (22)	NA
No response or skip	3 (11)	NA
As the COVID-19 pandemic is progressing, what are the most pressing health issues for you?^b^		
Long COVID-19	3 (11)	NA
Getting or spreading COVID-19	7 (26)	NA
Mental health	9 (33)	NA
Physical health	6 (22)	NA
Not informative or other	3 (11)	NA
No response or skip	2 (7)	NA
Week 3 survey questions: Mental well-being (n = 25 respondents)		
What does health and well-being mean to you?^b^		
Good mental health	12 (48)	NA
Good physical health	13 (52)	NA
Not informative or other	4 (16)	NA
No response or skip	5 (20)	NA
How are you doing, really?^b^		
Poor mental health	3 (12)	NA
Ok mental health	8 (32)	NA
Good mental health	5 (20)	NA
Improving	6 (24)	NA
Not informative or other	4 (16)	NA
No response or skip	1 (4)	NA
What factors contribute the most uncertainty or anxiety in your life, if at all?^b^		
Financial stress	2 (8)	NA
Work or employment	2 (8)	NA
School	9 (36)	NA
Social life or peers/family	9 (36)	NA
Not informative or other	6 (24)	NA
No response or skip	2 (8)	NA
Week 4 survey questions: Risk-taking during COVID-19 (n = 25 respondents)		
How often did you eat outdoors?		
Whenever I felt like it	8 (32)	13 (52)
Only when others invited me	4 (16)	5 (20)
Only for special occasions	11 (44)	5 (20)
Never! (Even if my best friend invited me)	2 (8)	2 (8)
Prefer not to answer	0	0
How often did you go to the movies?		
Whenever I felt like it	4 (16)	11 (44)
Only when others invited me	8 (32)	4 (16)
Only for special occasions	3 (12)	5 (20)
Never! (Even if my best friend invited me)	9 (36)	5 (20)
Prefer not to answer	1 (4)	0
How often did you go to a social gathering with more than 10 people?		
Whenever I felt like it	0	9 (36)
Only when others invited me	10 (40)	8 (32)
Only for special occasions	10 (40)	7 (28)
Never! (Even if my best friend invited me)	5 (20)	0
Prefer not to answer	0 or	1 (4)
How often did you hang out with friends unmasked?		
Whenever I felt like it	8 (32)	16 (64)
Only when others invited me	5 (20)	4 (16)
Only for special occasions	6 (24)	4 (16)
Never! (Even if my best friend invited me)	5 (20)	1 (4)
Prefer not to answer	1 (4)	0
How often did you leave your current state?		
Whenever I felt like it	5 (20)	11 (44)
Only when others invited me	1 (4)	1 (4)
Only for special occasions	11 (44)	6 (24)
Never! (Even if my best friend invited me)	8 (32)	6 (24)
Prefer not to answer	0	1 (4)

Abbreviation: NA, not applicable.

^a^ Table data are unweighted.

^b^ Denotes an open-ended question.

Twenty-seven of 49 participants (55%) completed the week 2 survey. When asked to identify the top issue for improving community health, 19% highlighted increased access to health care and health insurance, although many responses were not informative. Additionally, 59% of respondents were not part of any health-supportive organizations, whereas 26% were part of 1, and 15% were part of more than 1. Community events and educational workshops were seen as the most helpful resources to improve health, whereas mental health, COVID-19, and physical health were identified as the most pressing health concerns.

Twenty-five of 49 participants (51%) completed the week 3 survey. Although 48% of respondents linked good mental health to overall well-being, only 20% indicated that their mental health was good, with many reporting anxieties related to school and social relationships.

Twenty-five of 49 participants (51%) completed the week 4 survey. Respondents reported increased risk-taking behaviors from the height of the COVID-19 pandemic to 2023. The proportion of people eating indoors increased from 32% in 2020 and 2021 to 52% in 2023, and those attending large social gatherings increased from 0% to 36%. Similarly, unmasked gatherings increased from 32% to 64%, and travel outside of their home state increased from 20% to 44%.

## Discussion

This survey study highlights that mental health is a major concern among youths from historically marginalized racial and ethnic groups in Boston during the postacute phase of the COVID-19 pandemic. Survey findings suggest that increasing the availability of health-supportive organizations, online content tailored to youth concerns, and in-person health education outreach events could help address this population’s needs. Through collaboration with youths in survey design, the study gained valuable insights and created opportunities for young leaders from marginalized communities to actively engage in public health research. Limitations of this study include sample size, participant attrition, and administrative constraints on incentive distribution. Refining the incentive structure and extending the survey period could enhance engagement and allow this instrument to more effectively reach and support this population in future studies.

## Notes

Supplement 1.
eMethods

Supplement 2.
Data Sharing Statement
