# Supplementary material for: Health Concerns of Youths From Historically Marginalized Communities During the Postacute Phase of COVID-19
Source: JAMA Netw Open. 2025 Mar 14;8(3):e250837. doi: 10.1001/jamanetworkopen.2025.0837 (PMC11909605; doi:10.1001/jamanetworkopen.2025.0837)
Supplement: Supplement 1. — eMethods [file jamanetwopen-e250837-s001.pdf]

Supplemental Online Content

Jones MA, Jones M, Mazor KM, Linas BP. Health concerns of youths from underrepresented communities during the postacute phase of COVID-19. *JAMA Netw Open*. 2025;8(3):e250837. doi:10.1001/jamanetworkopen.2025.0837

eMethods

This supplemental material has been provided by the authors to give readers additional information about their work.

© 2025 Jones MA et al. *JAMA Network Open*.

eMethods

Detailed Methods Overview

Survey Development

The research team developed survey items with the goal of exploring key questions that would provide an understanding of participants' health concerns and elucidating mechanisms to best address them. After creating a draft of themes of interest, questions for each theme were developed. The questions and themes were then presented to a group of 8 high school students, who provided feedback on question wording and content. Questions were revised to incorporate this feedback. A larger group of high school and college students involved with WGU, alongside members of the CEAL team were invited to partake in a trial-run of the survey. The research team revised questions based on their feedback. The resulting questions comprised the final version of the survey questions.

Survey Implementation

A key goal in survey implementation planning was to maximize access, participation, and retention. To achieve this, surveys were designed to be brief, each taking approximately 5 minutes to complete, and were distributed as a series of 5 short surveys over 5 consecutive weeks, rather than a single lengthy survey. Additionally, participants were organized into cohorts based on their enrollment date to ensure prompt engagement after recruitment, with each cohort having a 2-week enrollment period.

Each weekly survey was programmed in REDCap, with the survey link delivered via text message. All weekly surveys were sent at the same time each week and remained open for 6 days

© 2025 Jones MA et al. *JAMA Network Open*.

and 22 hours. Reminder texts were sent to participants who had not yet completed the surveys, with the first reminder sent 3 days before the survey closed and a second, if needed, 24 hours before the deadline.

Incentives

Eligible participants earned $5 after completing the demographic survey, an additional $1 for each weekly survey completed, and $5 at the end of the study if all five surveys were fully completed. Incentives were delivered using Amazon e-gift cards, which were sent to participants via email at the end of the study period. This incentive method was chosen based on the anticipation that it would appeal to younger participants and was in line with the methodology of digital distribution of materials to participants.

Data Cleaning

After the demographic survey was completed, the survey responses were manually screened twice for quality assurance. Entries that appeared to be computer-generated were removed using a standardized checklist that identified patterns based on start time, duration, email, and responses to free response questions. A reCAPTCHA feature was included in the REDCap survey link to increase security.

Data Analysis

Data from all five cohorts were pooled and analyzed as one group. Conventional content analysis was used to analyze responses to open-ended questions, identifying key concepts in the

© 2025 Jones MA et al. *JAMA Network Open*.

responses, applying codes to categorize the presence of these concepts in each response, and then organizing the codes into overarching themes.

In the first step, two researchers analyzed the responses to each of the open-ended survey questions and identified key concepts in the responses for each question. Then, each researcher independently reviewed the responses and generated an initial set of codes that correlated to these concepts. The two researchers then met and compared the initial set of codes and made modifications as needed. Results from this preliminary round of coding were then discussed with an additional researcher not involved in the coding process, who provided guidance on the formation of new categories and additional modifications to existing categories. A second round of coding was then completed independently by both researchers, after which, agreement of final codes for each response was identified.

© 2025 Jones MA et al. *JAMA Network Open*.
