# Supplementary material for: Health Concerns of Youths From Historically Marginalized Communities During the Postacute Phase of COVID-19
Source: JAMA Netw Open. 2025 Mar 14;8(3):e250837. doi: 10.1001/jamanetworkopen.2025.0837 (PMC11909605; doi:10.1001/jamanetworkopen.2025.0837)
Supplement: Supplement 2. — Data Sharing Statement [file jamanetwopen-e250837-s002.pdf]

Data Sharing Statement

Jones. Health Concerns and Behaviors of Racial and Ethnic Marginalized Youth During the Postacute Phase of COVID-19. *JAMA Netw Open*. Published March 14, 2025. doi:10.1001/jamanetworkopen.2025.0837

Data
Data available: Yes
Data types: Deidentified participant data
How to access data: Deidentified data will be made available from the corresponding author upon request (melissajones@college.harvard.edu/majones.sci@gmail.com).
When available: With publication

Supporting Documents
Document types: None

Additional Information
Who can access the data: Data will be made available to researchers whose proposed use of the data has been approved.
Types of analyses: Data will be made available for research pertaining to social determinants of health and youth health disparities.
Mechanisms of data availability: Data will be made available with a signed data access agreement.
Any additional restrictions: N/A
